# Automated Pipeline for Robust Cat Activity Detection Based on Deep Learning and Wearable Sensor Data

**DOI:** 10.3390/s24237436

**Published:** 2024-11-21

**Authors:** Md Ariful Islam Mozumder, Tagne Poupi Theodore Armand, Rashadul Islam Sumon, Shah Muhammad Imtiyaj Uddin, Hee-Cheol Kim

**Affiliations:** 1 Institute of Digital Anti-Aging Healthcare, Inje University, Gimhae 50834, Republic of Korea; arifulislamro@gmail.com (M.A.I.M.); poupiarmand2@gmail.com (T.P.T.A.); sumon39cst@gmail.com (R.I.S.); imtiyaj.dream@gmail.com (S.M.I.U.); 2 Department of Computer Engineering, Inje University, Gimhae 50834, Republic of Korea

**Keywords:** activity detection, biosensors, deep learning, CNN, pet activity

## Abstract

The health, safety, and well-being of household pets such as cats has become a challenging task in previous years. To estimate a cat’s behavior, objective observations of both the frequency and variability of specific behavior traits are required, which might be difficult to come by in a cat’s ordinary life. There is very little research on cat activity and cat disease analysis based on real-time data. Although previous studies have made progress, several key questions still need addressing: What types of data are best suited for accurately detecting activity patterns? Where should sensors be strategically placed to ensure precise data collection, and how can the system be effectively automated for seamless operation? This study addresses these questions by pointing out whether the cat should be equipped with a sensor, and how the activity detection system can be automated. Magnetic, motion, vision, audio, and location sensors are among the sensors used in the machine learning experiment. In this study, we collect data using three types of differentiable and realistic wearable sensors, namely, an accelerometer, a gyroscope, and a magnetometer. Therefore, this study aims to employ cat activity detection techniques to combine data from acceleration, motion, and magnetic sensors, such as accelerometers, gyroscopes, and magnetometers, respectively, to recognize routine cat activity. Data collecting, data processing, data fusion, and artificial intelligence approaches are all part of the system established in this study. We focus on One-Dimensional Convolutional Neural Networks (1D-CNNs) in our research, to recognize cat activity modeling for detection and classification. Such 1D-CNNs have recently emerged as a cutting-edge approach for signal processing-based systems such as sensor-based pet and human health monitoring systems, anomaly identification in manufacturing, and in other areas. Our study culminates in the development of an automated system for robust pet (cat) activity analysis using artificial intelligence techniques, featuring a 1D-CNN-based approach. In this experimental research, the 1D-CNN approach is evaluated using training and validation sets. The approach achieved a satisfactory accuracy of 98.9% while detecting the activity useful for cat well-being.

## 1. Introduction

Recognition of activity and the analysis of diseases of different types of pets and humans has been increasing simultaneously in recent years. For their cuteness and kindness, cats are the most popular choice for household or outdoor pets. Recently, primitive commercial services have started to record cats’ activities and report them to their owners or health instructors. It is vital to assess the activity of undisturbed animals to properly comprehend their ecosystem or offer indices of their welfare, but this is a difficult undertaking [1]. Multiple physical conditions and physiological factors may be monitored simultaneously with the position of the animals, due to the inclusion of numerous sensors in transmitters [2]. Due to recent advances in data loggers that capture body movements using acceleration signals, it is possible to develop and install a variety of animal sensors, allowing researchers to monitor varied behaviors [3,4,5]. In addition to direct observation by humans, the rapid development of digital information-processing technologies provides a lot of chances to investigate animal behavior through more accurate activity analysis [6]. There is no secret in the world of bio-signal processing, because understanding data from several sensors linked to one’s body results in high precision and accuracy [7,8,9,10,11]. Automated systems for the detection of behaviors have become increasingly popular in recent years, owing to the ability of the associated sensors in the automated systems to discriminate between various activity patterns. Research has demonstrated that combining data from multiple sensors can lead to more effective and efficient activity detection processes [12,13,14]. There are many aspects to consider while making decisions about one’s health, state of mind, and activity, and hence utilizing several body sensors is thought vital in this scenario. However, throughout the last decade, the use of various body sensors to resolve a specific decision was a point of contention due to a lack of resources and effective frameworks for multi-sensory data handling. The process of quick prototyping and deployment has grown considerably easier with the introduction of body sensor networks (BSNs) and business cloud architectures. Body activity types are important for the identification of behavior patterns.

Consequently, accelerometers, gyroscopes, and magnetometers are generally used in automated systems. Accelerometers are small sensors that use micro-machined structures to measure acceleration along three axes denoted by the letters X, Y, and Z. When the sensor moves in a specific direction, it returns a value in meters per second squared for how fast it traveled along all its axes. Behavior such as resting and walking can be identified efficiently using accelerometer sensor data. Cat Activity Recognition (CAR) is classifying the activity of a cat using responsive sensors that are affected by cat movement. The gyroscope’s ability to gauge rotational speed around a specific axis helps it continue to function as intended. In our study, the gyro sensor works with three axes (X, Y, and Z) to measure the activity rate, combined with the accelerometer. On the other hand, the magneto sensor also works with three axes to measure the angular rate, combined with the accelerometer. According to previous studies [15,16,17,18,19,20,21,22,23,24,25], systems are implemented and evaluated with the use of deep learning methods, namely, Artificial Neural Networks (ANNs), Long Short-Term Memory (LSTM), and a One-Dimensional Convolutional Neural Network (1D-CNN). Most of these past studies focused on traditional machine learning techniques for the activity detection of different animals using wearable accelerometers and some gyroscopes. Simultaneously, a few other researchers looked at accelerometer data for activity, but very few studies have looked at the combination of accelerometer, gyroscope, and magnetometer sensors for activity detection and classification. We opted to employ neck-wearable sensors to detect the activity patterns of domestic pets after gathering a lot of evidence from previous studies.

In our research, we attempted to create an automated system that analyzes data from neck sensors to classify activity patterns. For the performance comparison, we used standard deep learning approaches as well as incremental machine learning techniques. We also evaluated our system using validation sets of data, and the machine learning models provided the best prediction performance, indicating that our system has the potential to be employed in real-world scenarios. Finding a strong solution is the primary goal of this study, using deep learning algorithms for monitoring, detecting, and classifying the activities of domesticated cats, and carrying out an activity analysis of home-kept cats by leveraging the 1D-CNN-based deep learning approach. More specifically, we use a computer-assisted methodology for the classification of different types of activities.

Hence, the objectives of this study are as follows:

- To develop an automated system that can accept accelerometer, gyroscope, and magnetometer multi-axis data as input and distinguish different cat activity patterns by applying a deep learning algorithm.
- With the incorporation of multi-sensor devices on cats’ necks, to use feature engineering for best real-time accuracy.
- To address imbalance issues by employing the class weight approach, and to carry out hyperparameter tuning to achieve the optimal performance of the model.
- To test the automated system developed using a 1D-CNN model to accurately detect cat activities in real time.

## 2. Related Work

Multiple activities are detected among humans and animals, especially dogs and cats, using sensor data. Many researchers have used machine learning for this purpose, but the trend has been changing in the last few years from machine learning to deep learning. Because deep learning models give more accurate and better results compared to machine learning models, they facilitate the detection and classification of multiple activities among dogs which are very important for their health and fitness [26].

Previous research by Hussain et al. used sensors worn on the collars of dogs to acquire the data, and their created framework was able to determine 17 different actions of 18 dogs with results of almost 70% accuracy [27]. Sanhudo et al. used a tri-axial accelerometer and gyroscope, mounted on the backs of dogs, to analyze activities and behavior based on simultaneous video recordings. Their study was able to check the feasibility of wearable devices for activity detection among dogs but was not validated in real-life situations [28]. Aich et al. proposed an automatic model to detect the activity and emotions of dogs using machine learning classifiers including Support Vector Machines (SVMs), Naïve Bayes, K-Nearest Neighbors (KNN), and an ANN for activity detection. Among these machine learning classifiers, the ANN outperformed the rest [29]. Moreso, a model that can determine the behavior of dogs based on accelerometers for data collection from 51 different dogs of different ages, weights, and breeds was proposed by Maza et al. [30]. The proposed model obtained an overall accuracy of 95%. Chakraborty et al. used smart sensing devices to determine the activities and emotions of animals. They used three different sensors including temperature, galvanic, and ECG in their study for data collection. In their study, they predicted four different emotions in animals: happiness, sadness, anger, and neutral [31].

Furthermore, Kiyohara et al. showed a model to determine the actions and behaviors of moving dogs. They used supervised machine learning in their study and, for data collection, they used multi-sensor logger devices. In their study, they also considered the battery timing for long-time activity detection in dogs [32]. Vehkaoja et al. used deep learning techniques to determine activities among different dogs [33]. Hussain et al. used similar types of sensors for data collection. In their deep learning-based model, they trained a Convolutional Neural Network (CNN) model. After testing this model, they compared the performance with other traditional models. After comparison, they found that their model was sufficiently reliable for activity detection. A LSTM model was trained and installed on different wearable sensors [34]. Hussain et al. developed a 1D-CNN-based model for detecting dog activity using sensor data. Their model could classify ten different activities of dogs. The data was collected from 10 dogs of different breeds, ages, sizes, and genders. They preprocessed the data before it was used for the training of the model. They used 80% of the data for training and 20% for the testing of the model. The model achieved a training accuracy of 99.70% and a validation accuracy of 96.85% [35].

Venkatraman et al. illustrated and used very small sensor devices for small animals like rats. These small devices catch the acceleration data from animals in cages when they act naturally. By using neural network-based pattern recognition algorithms, they predicted the behavior of the animals. Three basic actions were successfully identified, including grooming, standing, and feeding. The research achieved an accuracy of almost 98% for grooming, 97% for standing, and 93% for feeding activity [36]. Yen et al. proposed a deep learning-based model to determine six types of different human activities: walking, walking downstairs, walking upstairs, lying, standing, and sitting. They used a gyroscope, and an accelerometer mounted on the waist of the human body to acquire the data. They trained a 1D-CNN on two different datasets: the University of California dataset and their own recorded dataset. They achieved almost 96% testing accuracy on the UCI dataset and 93% testing accuracy on their dataset [37]. Axiu et al. carried out a comparison analysis of animal activity detection from wearable sensors based on deep learning [38]. Minati et al. introduced an approach to time series data augmentation involving driving a single low-dimensional entity, namely, the Rössler system, with a physically recorded sensor signal, and leveraging its responses to enhance the performance of a conventional classifier [39].

Table 1 lists the earlier work on the wearable sensor-based activity detection of various pets. The research cited in Table 1 illustrates how wearable devices, particularly accelerometers, gyroscopes, and Photoplethysmogram (PPG) and Electrocardiogram (ECG) sensors, have been used to detect behavioral patterns in animals, including activity detection. However, we have observed that previous studies have mostly employed accelerometer and gyroscope data to detect pet activities. Except for a very small number of experiments, most did not explore the simultaneous use of accelerometer, gyroscope, and magnetometer data for pet activity detection.

## 3. Materials and Methods

In this section, we are going to describe the methodologies that are used in this research, including information about cats, sources of data, feeding and husbandry environment, and physical sensors.

### 3.1. Data Acquisition

#### 3.1.1. Cats

The total number of cats was 10. Among those, 4 were males and 6 were females with different ages, sizes, and breeds. The cats were healthy and in good condition during the experimental period. Table 2 below shows the details about the experimental cats.

#### 3.1.2. Source of Data

The data were collected from 10 cats from 1 November 2021 to 30 November 2021. The dataset contained 1,284,789,349 samples of tri-axial data from accelerometer, gyroscope, and magnetometer sensors. We took the cats’ real-time data from Ujura Company (Seoul, Republic of Korea) and we used it for our experiment on cat activity detection.

#### 3.1.3. Husbandry

**Figure 1:**
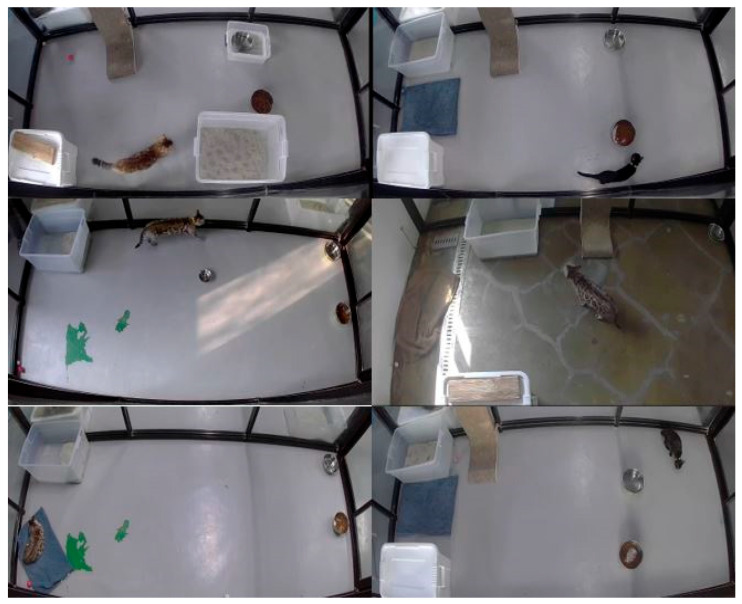
Housing, monitoring, and husbandry environment of the cats.

The cats were guided by the expertise of husbandry and veterinary professionals; they designed a diet that catered to their health needs. Their living place was very clean, and a spacious environment with proper lighting was provided for all the cats. The rooms were 4.0 m × 3.5 m in size. To provide a better environment and to keep them active, the cats were provided with balls to play with. For the scratching of the cats, proper rugs were placed in each room. In every room, a box filled with water was provided for drinking purposes in case the cats were thirsty, and a different box was kept likewise for urinating and defecating. Figure 1 shows the experimental environment of the cats.

#### 3.1.4. Sensor Device

**Figure 2:**
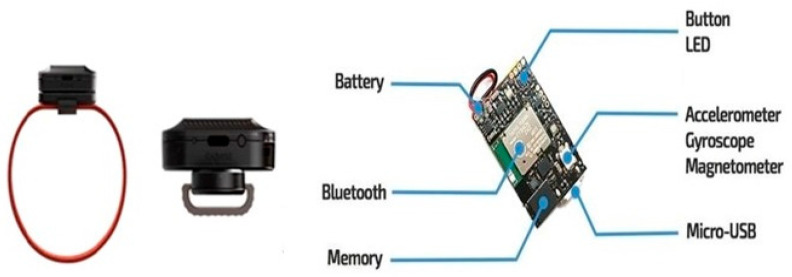
Wearable sensors with internal features.

The wearable devices consisted of three sensors, namely, accelerometer, gyroscope, and magnetometer sensors, and one device was placed on the neck of each cat. These sensors were able to measure the linear motions, rotational motions, and magnetic motions in all three axes, i.e., x, y, and z. The gyro sensor has a range of ±2000 DPS and a sampling rate of 0.001–100 Hz, and the accelerometer has a range of ±16 g and a sampling rate of 0.001–100 Hz. Likewise, the magnetometer has a range of ±1300 μT (*x*, *y*-axis), ±2500 μT (*z*-axis), and a sampling rate of 0.001–25 Hz. The devices weighed 0.2 oz and the dimensions were 27 mm × 27 mm × 4 mm. The sensor devices are designed to detect cat movements through rotational, linear, or magnetic motions. The data were timestamped for synchronization purposes. Each device has a Lipo battery with 70–100 mAH and a charging time of 2 h. Figure 2 shows the internal details of the sensor and the outfit of the sensor.

#### 3.1.5. Video Recording

The activities of cats were recorded through CCTV cameras as well. The collected data from the sensor devices were synchronized with the video for the ground truth and to ensure the correct labeling of different activities. Ten researchers classified the videos using manual classification, and their work was verified by the senior specialist and CTO from Ujura Company, Republic of Korea.

#### 3.1.6. Data Collection

**Figure 3:**
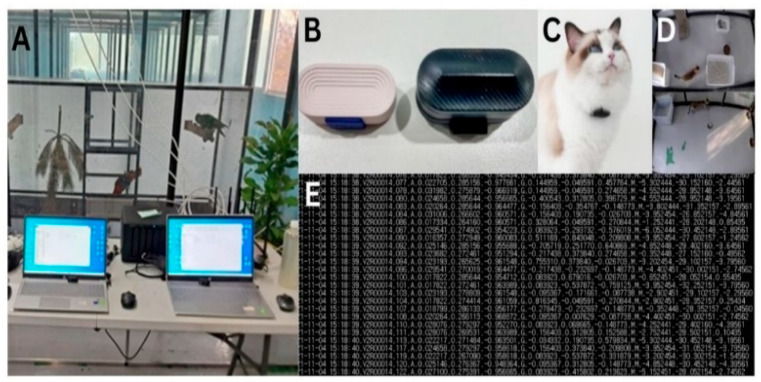
Data collection procedure. (**A**) Server room for real-time monitoring and storing data, (**B**) sensor device, (**C**) sensor device on the cat’s neck, (**D**) cat living space, including surveillance cameras, (**E**) transferring sensor data to the server.

The data used in this research were collected with prior authorization from the cats’ owner (Ujura Company). The data were generated by the movement of the cats, and three kinds of data have been generated: linear motion data from the accelerometer, rotational motion data from the gyroscope, and magnetic motion data from the magnetometer sensors. The data were sent to the server via Bluetooth and were stored on the server. At the same time, video recording was also performed for the corresponding sensor data, and the sensor data were labeled while using the video recording. In Figure 3, we show all the steps of the data collection procedure.

The data were processed and analyzed using a system with the following specifications: Windows 11, 2.50 GHz 64-bit, 12th Gen, Intel Core i5-12400 processor, 32 GB RAM, NVIDIA GeForce RTX 3080 GPU, Python 3.9, and TensorFlow 2.4.0.

**Figure 4:**
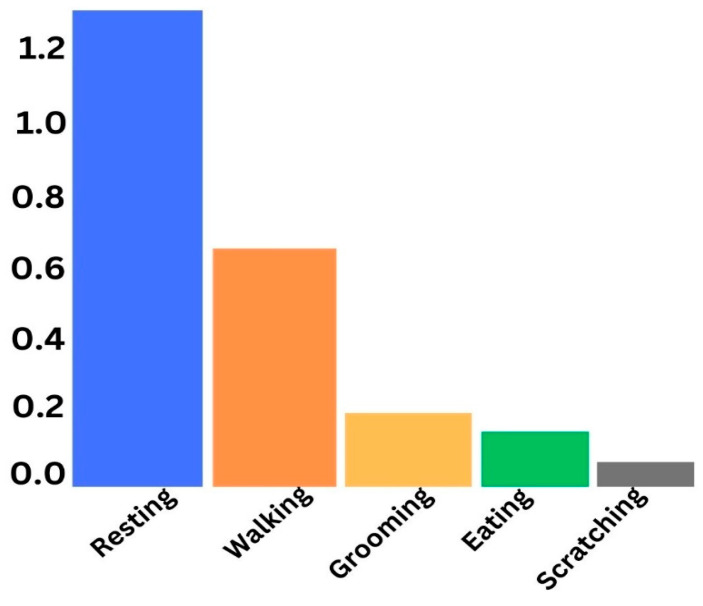
Data distribution of activity detection.

**Figure 5:**
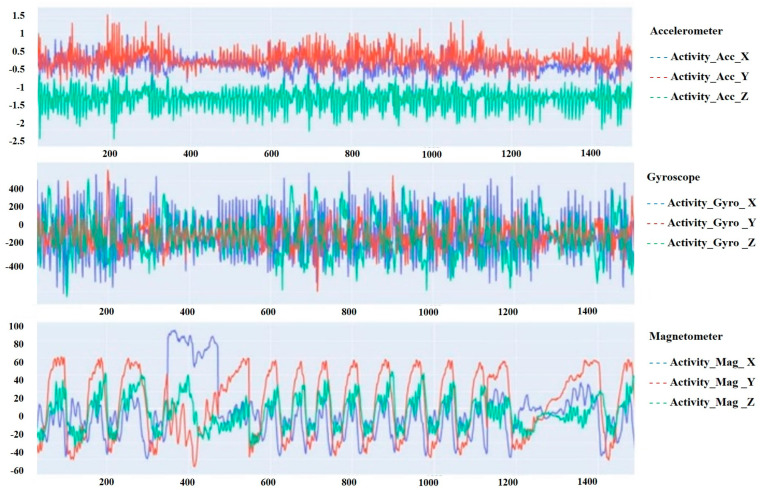
Samples of bio-signals from the wearable devices on the cats.

Figure 4 shows that the data distribution across all the five classes is highly imbalanced, so this behavior of data may render the model overfitting. This issue must be addressed before the data are used for the training of the model. Figure 5 below represents a sample of the bio-signals obtained from the sensor devices attached to the cats.

#### 3.1.7. Preprocessing of the Data

This process is vital because sensor data is often noisy, contains missing values, and requires proper formatting before it is fed it into machine learning models for activity detection tasks [46]. We take the raw sensor data, which is initially labeled, and then proceed to enhance its quality and prepare it for training in an artificial intelligence model. This involves crucial steps to handle noise and anomalies that might be present in the data, particularly in the bio-signals reflecting the cats’ activities. To address the issue of noisy data, we employ the Butterworth low-pass filter, effectively eliminating unwanted high-frequency noise components while preserving the essential bio-signals. This ensures that the actual activities of the cat are accurately represented in the processed data. By refining and cleansing the data through these smart preprocessing techniques, we have developed a robust and accurate dataset suitable for training the machine learning model. This essential step significantly enhances the model’s ability to interpret the cats’ activities and paves the way for more reliable artificial intelligence predictions.

#### 3.1.8. Feature Engineering

To extract valuable insights from the sensor data, we applied feature engineering techniques. By analyzing accelerometer, gyroscope, and magnetometer data, we derived key features, most important features such as standard deviation, mean absolute deviation, mean, minimum, maximum, interquartile range, energy measure, skewness, kurtosis, etc. Zheng et al. have presented windowing techniques for activity detection using sensor devices, which involve segmenting the continuous stream of sensor data into smaller time windows or frames [47]. This windowing technique was used to divide the data into 2 s windows with 50 data samples per window, overlapping 25 samples from the previous window. This allowed us to create new features while preserving temporal information. The transformed data were labeled based on the most frequent activity within each sliding window. We applied a fast Fourier transform to convert time domain data into the frequency domain, gaining deeper insights and enriching the dataset. In the end, our feature engineering efforts yielded 312 informative features. Kempa-Liehr et al. showed that the goal of feature engineering is to provide algorithms with informative input features that capture the essential patterns and characteristics of the activities, enabling accurate and efficient activity detection and classification [48,49].

#### 3.1.9. Class Weight Approach

There are different approaches for balancing the data, for example, Random Oversampling, the synthetic minority oversampling technique (SMOTE), Adaptive Synthetic Sampling (ADASYN), etc. [50]. To keep a balance among the classes, a threshold should be defined so that class weights can be increased or decreased. With this technique, we take more care of the minority samples while training the model and, to calculate the loss function, a weighting mechanism is developed. Different weights are assigned to majority and minority classes according to the imbalance scenario in the dataset. To keep a balance among the classes, a threshold should be defined so that class weights can be increased or decreased. This will help in preventing the biasing of the algorithm towards any specific class. The formula for class weight can be defined as

(1) $$W_{i}=\frac{n\text{\_}instances\;}{\;(n\text{\_}classes\;*\;{n\text{\_}instances}_{i})}$$

where ($W_{i}$) represents the weight of each class and (*i*) represents the class. *n_instances* denotes the total number of instances or rows in our dataset, whereas *n_classes* represents the overall number of unique classes in the class label. The total number of rows in each class is denoted as ${n\text{\_}instances}_{i}$. The weighting mechanism adopted in this study is shown in Table 3 below.

### 3.2. Methods

#### 3.2.1. Proposed Activity Detection Algorithm

A One-Dimensional Convolutional Neural Network is a powerful algorithm for activity detection based on sensor data from wearable devices [51,52]. A proposed pet activity detection algorithm was developed, which included the collection of bio-signals from wearable devices, i.e., an accelerometer, a gyroscope, and a magnetometer. The bio-signals were preprocessed by applying different preprocessing techniques like data filtration, data normalization, etc. The activities of the cats were predicted using the CNN-based algorithm as a well-known deep learning approach used for different purposes like classification and detection. It automatically extracts the highly relevant features without any human intervention or handcrafted methods and uses those features for classification and detection purposes. We used sensor data consisting of x, y, and z values, and we transformed them into vector magnitude data. This vector was used to develop a 1D-CNN for the classification of the different activities of the cats.

#### 3.2.2. The Network Architecture of 1D-CNN

Convolutional Neural Networks (CNNs) have garnered widespread acclaim in the realm of deep learning due to their remarkable capabilities in various applications, such as classification and detection tasks. Unlike traditional Artificial Neural Networks, CNNs possess a unique ability to autonomously extract essential features from data without relying on manual intervention or handcrafted methods. This intrinsic feature extraction capability empowers CNNs to excel in both feature extraction and subsequent classification processes. In our research, we harnessed the potential of CNNs to explore the activities of cats using sensor data that comprised x, y, and z values. To facilitate a more efficient representation of the data, we transformed it into vector magnitude data. This transformed vector served as the basis for the development of a sophisticated 1D-CNN model tailored specifically for the classification of different feline activities. Our 1D-CNN architecture was thoughtfully designed, encompassing key components such as convolutional layers to learn intricate patterns, dropout layers for enhanced generalization and robustness, flattened layers to reshape the data for seamless processing, fully connected layers to establish meaningful connections, and SoftMax layers for accurate probability distribution across the various cat activities. By leveraging this comprehensive 1D-CNN model, we were able to gain deeper insights into the distinct activities exhibited by cats, paving the way for future advancements in understanding and analyzing animal behavior. The results of our study not only underscore the effectiveness of CNNs in the realm of activity classification but also shed light on the tremendous potential of deep learning architectures for pushing the boundaries of knowledge discovery and problem-solving in diverse domains.

**Figure 6:**
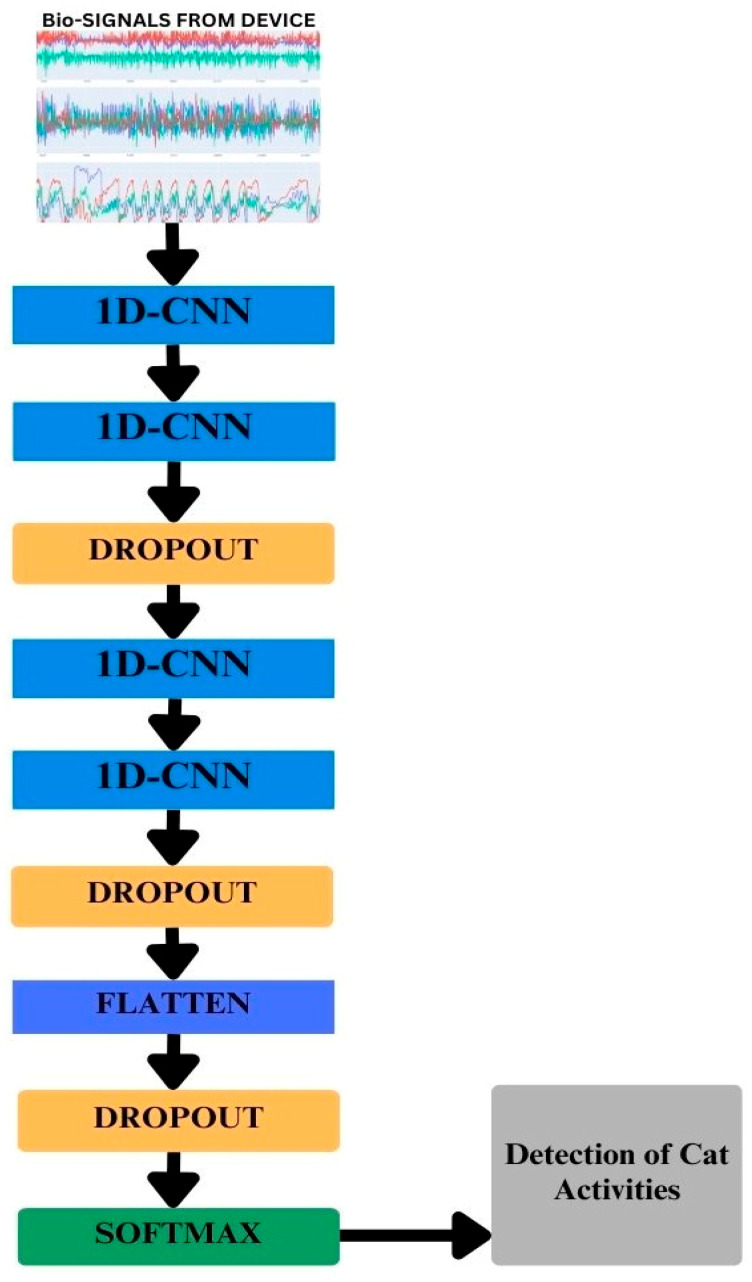
The deep learning model architecture of our experimental research work.

**Figure 7:**
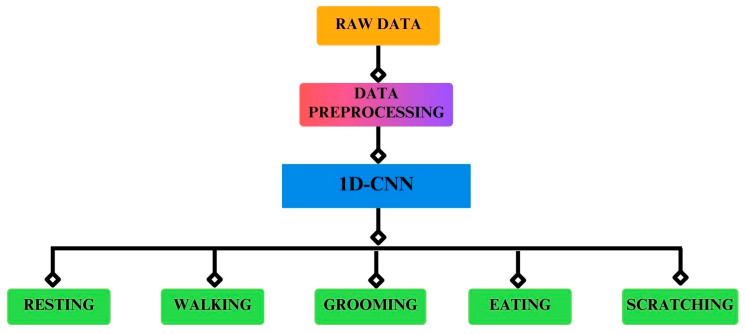
Classification of the five activities.

- Input layer: The input layer of the model received three-axis data from each accelerometer, gyroscope, and magnetometer sensor in the form of vector magnitude.
- Convolutional layer: The convolutional operations were used with a stride size of 1. The kernels used in the convolutional layers were 128, 128, 128, 256, and 256, while the strides were kept at 1 in each layer.
- Dropout: To avoid overfitting and to reduce the complexity of the model, dropout layers were used while the dropout value was set to 0.5.
- Output: In deep learning, activation functions play an important role in the prediction of any task. The right and wise choice of activation function results in good prediction. Rectified Linear Unit (ReLu) was used in this experiment. Since we had activities from five cats, which is a multiclass classification, we used the SoftMax function for the classification of all five activities. A Stochastic Gradient Descent (SGD) optimizer was applied, and the learning rate was set to 0.0001. Categorical cross-entropy was used as a loss function, which calculates the loss between the actual and predicted values. The smaller the difference between the values, the higher the performance of the model. Figure 6 illustrates the architecture of the model and Figure 7 shows the classification of the activities.

#### 3.2.3. The Proposed Research

**Figure 8:**
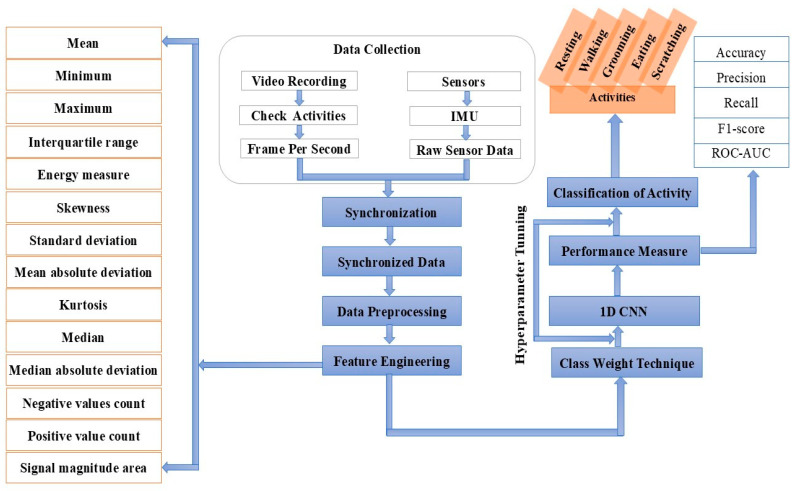
The complete process of the automated pipeline.

In our approach to detecting cat activities using data from wearable sensor devices, the overall process can be summarized as follows:

Firstly, we meticulously extracted data pertaining to five distinct activities and concurrently captured corresponding videos, synchronizing them at a precise frame rate for each activity. Subsequently, we undertook rigorous data preprocessing, effectively eliminating noise and unwanted bio-signals from the dataset. Employing a Butterworth low-pass filter, we successfully eliminated noise, thereby enhancing the dataset’s quality. To harness the full potential of the dataset, we engaged in feature engineering, a critical step that extracted pertinent information while discarding unnecessary data. This strategic maneuver facilitated the construction of an efficient algorithm, primed for accurate activity classification. Furthermore, we recognized the importance of data normalization to ensure that all data points fell within the same value range. The raw data extracted from the sensor devices were preprocessed and filtered by applying a Butterworth low-pass filter. The filters removed the noise and unwanted signals from the data and, as a result, we obtained refined data. Data normalization was applied to the dataset to normalize the range of all the data and bring them to the same scale. This process played a crucial role in optimizing the subsequent stages of our analysis. To evaluate the performance of our model, we diligently split the data into an 80% training set and, after preprocessing the dataset, a 20% testing set. Acknowledging the data’s inherent imbalance, we employed data oversampling techniques on the training dataset, ensuring a balanced representation of all classes. We leveraged the class weight technique, further enhancing the model’s sensitivity to minority classes during training. Next, we developed a sophisticated 1D-CNN model and diligently trained it using the class-weighted training dataset. As we prioritized performance, we continuously monitored the model during hyperparameter tuning, ensuring that any potential issues were swiftly addressed. The culmination of our efforts yielded promising results. The experimental outcomes showcased the model’s exceptional performance, with the class weight technique playing a pivotal role in enhancing accuracy and reliability. Overall, our approach not only demonstrated the efficacy of the 1D-CNN architecture but also highlighted the importance of thoughtful data preprocessing and the strategic handling of class imbalances in activity classification tasks. Figure 8 shows the complete process of the development of the activity detection systems for household pets.

## 4. Experimental Results with Discussion

The experimental results are discussed in detail in this section. We conducted experiments using class weights for our class labels to balance the activities of the cats.

### 4.1. Evaluation Methods

The performance of the model is based on accuracy, precision, recall, F-score, and ROC.

❖ Accuracy: accuracy states how close our nearest value is to the known value:

(2) $$Accuracy=\frac{TP+TN}{TP+TN+FP+FN}\times 100\%$$

❖ Precision: this is the fraction of relevant instances among the retrieved instances:

(3) $$Precision=\frac{TP}{TP+FP}\times 100\%$$

❖ Recall: this is the fraction of relevant instances that were retrieved:

(4) $$Recall=\frac{TP}{TP+FN}\times 100\%$$

❖ F1-Score: this is a way of combining the precision and recall of the model, and it is defined as the harmonic mean of the model’s precision and recall:
  (5) $$F\text{-}score=2\times \frac{Precision\times Recall}{Precision+Recall}\times 100\%$$
  where *TP* represents a true positive, *TN* is a true negative, *FN* is a false negative, and *FP* is a false positive. Precision indicates the degree of accuracy of the model in predicting the correct classification of activities. For instance, eating was positive, and all other activities of the cats were negative. In this scenario, the correct classification of eating is divided by the sum of the correct classification, and the incorrect classification of eating gives the precision value.

### 4.2. Evaluation Methods (Graphical)

The prediction outcome of a classification model is summarized in the confusion matrix. By displaying the number of predictions that were accurate and inaccurate for each class, it displays the model’s performance. It presents details regarding the model’s actual and expected classifications. The model properly predicts the values in the diagonals while misclassifying the values outside of the diagonals.

#### 4.2.1. Matrix

**Figure 9:**
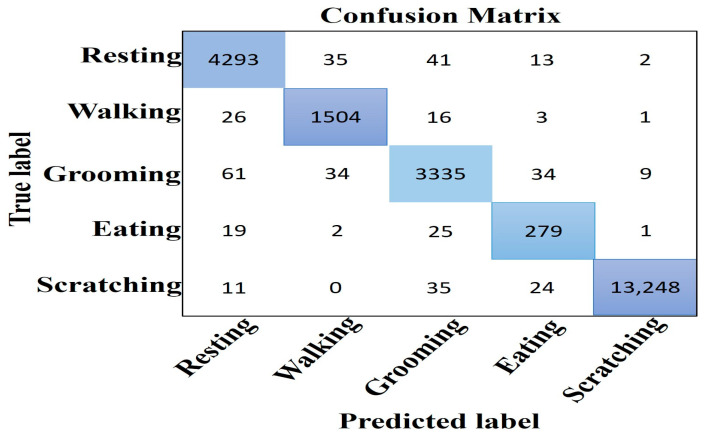
Confusion matrix without normalization using the test dataset.

**Figure 10:**
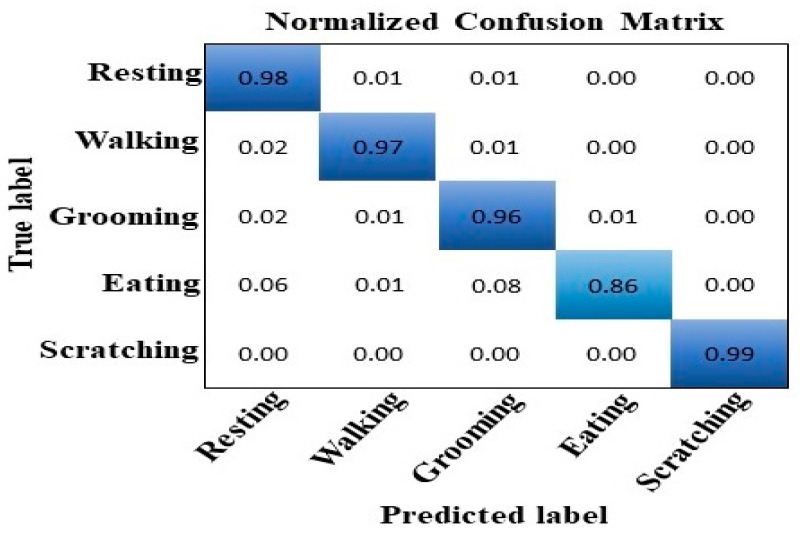
Confusion matrix with normalization using the test dataset.

The confusion matrices for all five of the behaviors of the cats studied in our research utilizing class weight are shown in Figure 9 and Figure 10, respectively, with and without normalization.

#### 4.2.2. Accuracy and Loss

**Figure 11:**
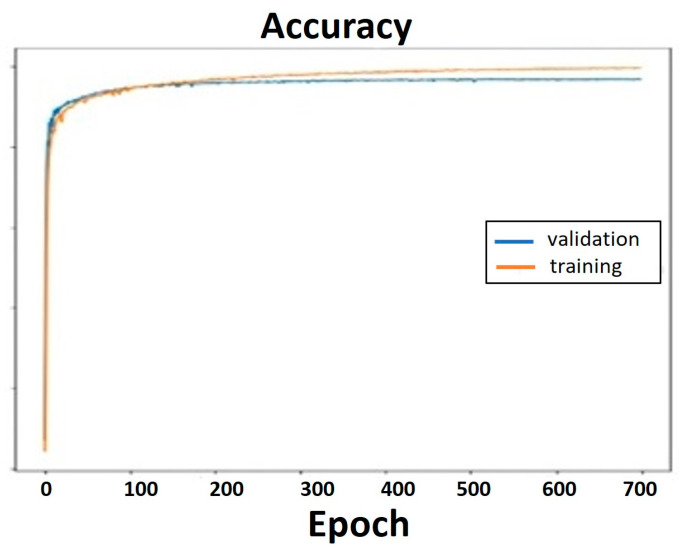
Accuracy graph for the validation and training.

**Figure 12:**
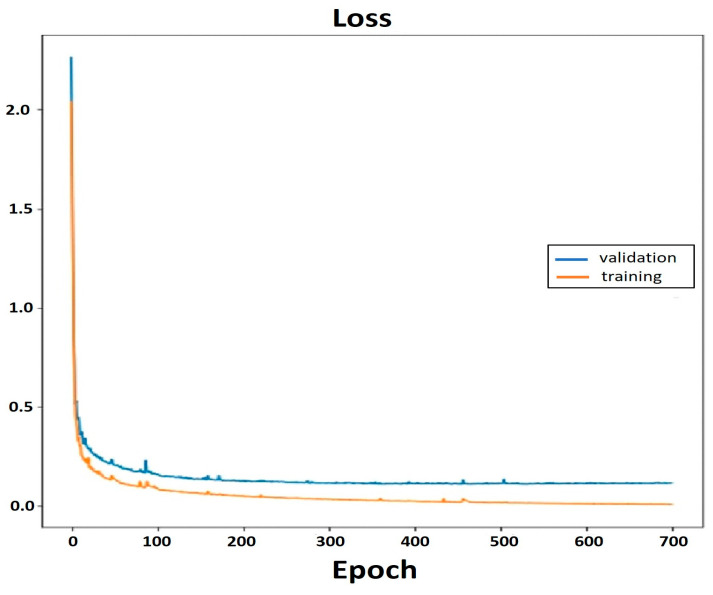
Loss graph for the validation and training.

Figure 11 and Figure 12 illustrate, respectively, the accuracy and loss of the model. Figure 11 demonstrates how the training accuracy rose to 98.9% after 700 epochs and 96.85% of the validations were accurate.

#### 4.2.3. AUC and RUC Curve

**Figure 13:**
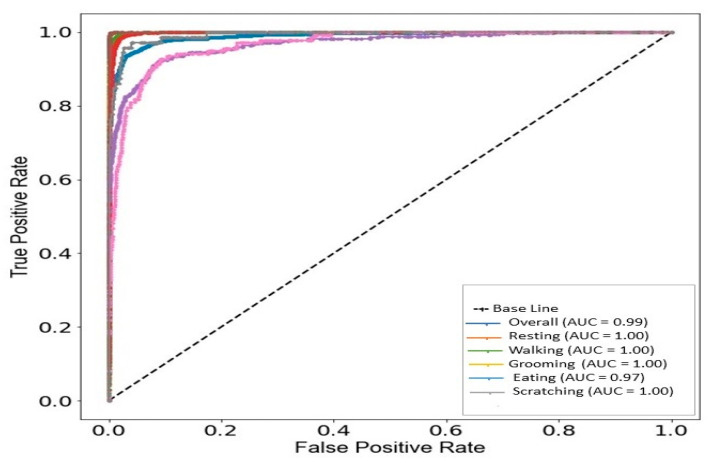
Receiver operating characteristic (ROC) curves and AUCs for each class.

The AUC-ROC curve helps us to visualize the performance of our proposed model. In other words, it is a measurement of evaluation that displays each class’s performance while drawing a graph between the true positive rate (TPR) and the false positive rate (FPR). The AUC-ROC is displayed in Figure 13. The better the model performs, the closer its graph is to the left corner and close to value 1. The graph below demonstrates that all the curves for each class are closer to 1, indicating that the model is performing at almost 100%. The AUC value of our suggested model is 100% for all five classes, demonstrating that our model correctly distinguishes between positive and negative class points.

## 5. Discussion

We have developed a highly efficient activity detection pipeline specifically designed for monitoring household pets through the utilization of wearable sensors attached to the neck. The pipeline incorporates data from accelerometers, gyroscopes, and magnetometers to extract various features. These features serve as input for accurately recognizing different activities, resulting in low misclassification rates.

Our findings underscore the intricacies involved in detecting the behavior of household pets. Notably, similar features extracted from diverse signal types prove effective in identifying various activities. Importantly, our model demonstrates impeccable performance. Although the accelerometer, gyroscope, and magnetometer data-based model has not been extensively studied for animals, a few research studies on humans, using different state-of-the-art techniques, yielded similar results to ours, establishing our proposed pipeline as state-of-the-art for animals, especially for cat activity detection.

This study introduces several novelties: (1) This is the first study to incorporate data from three different types of neck-wearable sensors—accelerometric, gyroscopic, and magnetometers for detecting activity patterns. This approach is particularly recommended for real-life situations involving pets as their activities, such as body movements involving the neck, are accurately captured using both types of sensors. (2) This study presents a robust pipeline utilizing an incremental learning-based deep learning algorithm for activity detection. The proposed activity detection system in this study outperforms previous research efforts. A comprehensive comparison with state-of-the-art models for activity detection is detailed in Table 4. We trained several machine learning and deep learning models; from the trained models, the 1D-CNN, the ANN, and LSTM have given the best results and, among these three, the 1D-CNN gave the best accuracy, as we have shown in Table 4. A 1D-CNN is ideal for cat activity detection as it excels in capturing temporal patterns in sequential sensor data, providing effective recognition of localized behaviors, and allows the efficient processing of wearable sensor information.

In this study, we collected data from a limited number of cats (10 cats). In the future, we will use more data for model training. This time, we worked on household cats; in the future, we will work on outdoor cats who live without limitations or borders. Also, in the future, we intend to focus on investigating activities separately and adding many more activities.

## 6. Conclusions

We introduced a 1D-CNN-based automated activity detection system to predict and classify five fundamental activities of cats. The data collected from cats of various ages, breeds, and genders were meticulously preprocessed and transformed into a suitable format for model training. Through feature engineering, we extracted the most meaningful and essential features from the raw data. Our proposed pipeline for detecting cat activities enables efficient monitoring of their well-being and overall health. This groundbreaking research proposes a 1D-CNN-based approach for cat activity detection, utilizing data from multiple sensors. We achieved a high accuracy of 98.9%, due to the integration of multiple sensors, which captures a comprehensive view of cat movements. This system holds great promise for enhancing our understanding of feline behavior and ensuring the welfare of our feline companions.

## Figures and Tables

**Table 1:** Related research on wearable sensor-based activity detection of various humans and animals.

Ref.	Sensors	Limitations	Key Results	Location	Pet Type
[29]	Tri-axial accelerometer and gyroscope	The classification accuracy of the model solely depends on the recording by the dog owner because that recording is used for annotation, which is not perfect. After all, manual error is inevitable. The proper environment has not been prepared for experimenting.	With an accuracy of close to 70%, the accelerometer data-based model can categorize various activities in natural environments.	Collar	Dog
[31]	Accelerometer, Electrocardiography and Electromyography	The work had flawed traditional accelerometer procedures. It was not used with tri-axial accelerometers.	Accelerometer and gyroscope data were analyzed based on simultaneous video recordings of different activities.	Wrist	Human
[35]	Tri-axial accelerometer and gyroscope	Activity detection for the well-being of household dogs using deep learning models from accelerometer and gyroscope data. Also, some of the dogs were very young.	Deep learning techniques were utilized to automate the system after acceleration and gyroscope data were used to identify the behavioral patterns of different dogs.	Neck	Dog
[36]	Tri-axial accelerometer	Performance comparison utilizing various machine learning techniques has not been explored and only acceleration data have been used to detect behavior.	To construct the algorithm to identify the rats’ activity patterns and learn more about their neurological actions, which aid in the detection of emotions, acceleration data were gathered using the sensors.	Back	Rat
[39]	Tri-axial accelerometer	Solely two species of chipmunks were utilized to create the model, which is insufficient from a validation standpoint, and the system only employed acceleration data to identify patterns.	Machine learning techniques were utilized to automate the system after acceleration data were used to identify the behavioral patterns of different chipmunk species.	Back	Chipmunk
[40]	Tri-axial accelerometer	Only accelerometer data have been considered for behavior detection, and the system has been evaluated using just one machine learning model.	To monitor the sheep’s behavior, the authors used accelerometer sensor data from different positions, and they achieved good results. They automated the system using machine learning techniques.	Collar	Sheep
[41]	Tri-axial accelerometer	Due to the lack of a state-of-the-art model, only accelerometer data were utilized to create the model, and the performance was not compared.	Acceleration data were utilized to identify the behaviors of different meerkat species, and hybrid methods (using machine learning and biomechanical principles) performed well.	Collar	Meerkat
[42]	Tri-axial accelerometer	They were wild animals, making this kind of analysis difficult. It is challenging to validate this model in real time.	Machine learning techniques were employed to classify the behavioral modes of the vulture using acceleration data and GPS data.	Back	Vulture
[43]	Tri-axial accelerometer, gyroscope	They used two different types of IMU sensors, an accelerometer, and a gyroscope to collect data. They focused on data augmentation techniques for behavior categorization. They did not deploy any machine learning or deep learning techniques for activity classification.	They used the data augmentation technique. Experimental results verify the data augmentation method’s effectiveness and show that their proposed behavioral monitoring method has greater advantages in terms of accuracy than traditional machine learning methods.	Neck	Pig
[44]	accelerometer and navigation satellite system (GNSS)	They used an accelerometer and a navigation satellite system (GNSS) on cattle collars and ear tags for collecting the data for activity classification. However, cattle are large-sized animals, so classifying the accurate activity of cattle is questionable. For big-sized animals, moving ears and collars do not mean that they are moving the full body.	They used MLP classifiers for classifying behavior and the multimodal animal behavior classification algorithm based on posterior probability fusion.	Collar and ear	Cattle
[45]	Tri-axial accelerometer	Only accelerometer data have been considered for behavior recognition, and the system has been evaluated using just one deep neural network model.	They applied deep learning for the activity recognition of individual hens, which has the potential to accurately aid the successful management of modern poultry systems.	Backpack	Chicken

**Table 2:** Experimental cats summary.

No.	Gender	Breed	Age in Years
1	Female	Siamese	2
2	Female	Chausie	3
3	Male	Maine Coon	4
4	Male	Maine Coon	5
5	Female	Maine Coon	5
6	Female	California Spangled	5
7	Female	Cornish Rex	6
8	Male	Abyssinian	7
9	Male	Chausie	7
10	Female	Toyger	7

**Table 3:** Class weights for activity detection model training.

Class	Weight
Resting	1.0516
Walking	2.9742
Grooming	1.3275
Eating	14.1211
Scratching	0.3461

**Table 4:** Class weights of three models for activity detection model training.

Metric		Classifier	
	ANN	LSTM	1D-CNN (Our)
Accuracy	0.9198	0.9615	**0.9896**
Precision	0.9188	0.9601	**0.9887**
Recall	0.9190	0.9625	**0.9891**
F1 Score	0.9181	0.9601	**0.9885**

## Data Availability

The internal dataset analyzed for this study is not publicly available at this moment. Data can be given upon request to the corresponding author.
